# Development of Best Practice Guidance on Online Peer Support for People With Young Onset Dementia: Protocol for a Mixed Methods Study

**DOI:** 10.2196/38379

**Published:** 2022-07-05

**Authors:** Esther Vera Gerritzen, Orii McDermott, Martin Orrell

**Affiliations:** 1 Institute of Mental Health Mental Health and Clinical Neuroscience, School of Medicine University of Nottingham Nottingham United Kingdom

**Keywords:** young onset dementia, peer support, eHealth, social health, mixed methods

## Abstract

**Background:**

Many people with young onset dementia (YOD) may feel isolated. Peer support has the potential to improve social health, but the inconsistent availability of age-appropriate, in-person (peer) support services for people with YOD suggests that many people with YOD miss out on the potential benefits. Online peer support could be useful, as it overcomes geographical barriers, offers a variety of options, and adjusts to various needs and preferences.

**Objective:**

Our study aims to develop evidence-based best practice guidance on online peer support for people with YOD and group facilitators to improve online peer support for people with YOD.

**Methods:**

Our mixed methods study consists of 4 phases and follows the guidelines of the Medical Research Council on complex interventions. Each phase consists of multiple substudies. The study focuses on the development stage of the Medical Research Council framework and additionally develops a plan for the feasibility/piloting, evaluation, and implementation stages. The participants are people living with YOD and peer support facilitators. The qualitative research methods include interviews, focus groups, and open questions in a web-based survey. The quantitative methods include a web-based survey consisting of existing outcome measures.

**Results:**

The study is funded by the European Union’s Horizon 2020 research and innovation program under the Marie Skłodowska-Curie Actions – Innovative Training Networks (H2020-MSCA-ITN-2018; grant agreement number: 813196), and it received ethical approval from the London Bromley Research Ethics Committee (reference number: 21/LO/0248) in April 2021. Recruitment started in May 2021. Data collection and analysis are expected to be finished by September 2022.

**Conclusions:**

The best practice guidance can provide people with YOD with tailored and evidence-based information about online peer support, and it will be disseminated locally (in the United Kingdom) and internationally through dementia organizations, research networks, and academic institutions.

**International Registered Report Identifier (IRRID):**

DERR1-10.2196/38379

## Introduction

### Young Onset Dementia

In 2018, around 1 million people were living with dementia in the United Kingdom, of whom almost 53,000 were younger than 65 years [1]. When someone is diagnosed before the age of 65 years, this can be defined as *young onset dementia* (YOD) [2]. People with YOD often face different challenges compared to those faced by older adults with dementia [3,4]. People with YOD are more likely to be in employment at the time of their diagnosis [5,6]. Difficulties at work and a YOD diagnosis can lead to (forced) early retirement, which has financial [7,8] and social [5,9] consequences, as this results in a loss of income as well as a loss of contact with colleagues. The loss of work can be perceived as a personal loss, as work is associated with involvement in meaningful activities and one’s identity [9,10]. Furthermore, people with YOD are more likely to fulfill an active parenting role toward dependent children. Changes in family structures can be experienced as losing one’s identity as a parent and being a burden to the family [7,11]. Additionally, people with YOD often experience a decrease in their social contacts and report losing touch with friends [7], which can be the result of a lack of understanding of YOD in the wider society. Stigmatization can result in the avoidance of social situations, which increases the risk of social isolation and loneliness [5,9,12]. These findings illustrate the unique challenges that people with YOD face when compared to those of older adults with dementia.

### The Social Health Framework

The social health framework looks at health within the social domain and includes the following three dimensions: (1) the ability to fulfill potential and obligations, (2) the ability to manage life with some level of independence, and (3) the ability to participate in social activities and work [13]. Dröes et al [14] suggest that when focusing on coping strategies and finding a balance between limitations and the abilities that one still has, people can adapt to living with dementia and still live meaningful and satisfying lives.

Peer support could improve all 3 dimensions of social health; hence, the social health framework will be used as the theoretical foundation for our study. Peers are people who have similar life experiences or health conditions [15,16]. First, peer support can be a way for people with YOD to stay socially connected and reduce the risk of isolation [17]. Besides offering a space for social connection, peer support creates opportunities to be involved in a variety of activities, such as creative and music-related activities, or with advocacy, research, and policy making, thereby allowing people to choose something that is meaningful to them. This relates to the dimension “the ability to participate in social activities and work” [13]. Second, through peer support, people can receive and provide support and share the unique knowledge that they have because of their own personal experiences of living with YOD. This knowledge is also called *experiential knowledge* and can include tips and tricks on how to manage dementia in daily life as well as information about support services [18,19]. This relates to the dimension “the ability to manage life with some level of independence” [13]. Third, the reciprocal nature of peer support and the opportunity to support others can increase feelings of empowerment [15,19,20]. This relates to the dimension “the ability to fulfill potential and obligations” [13]. Moreover, the work of Rabanal et al [21] and Stamou et al [22] shows that peer support can make the postdiagnostic experience more positive and can help people with YOD identify age-appropriate support services.

### Online Peer Support

People with YOD often experience difficulties in accessing local, age-appropriate support services, including opportunities for peer support [23,24]. Mayrhofer et al [23] show that support services for people with YOD vary widely across the United Kingdom. Additionally, services are often short-term because of project-based funding or because of services being offered as part of pilot studies [23], making it difficult for people with YOD to locate long-term, local, and age-appropriate (peer) support services [21]. As a result, a large group of people with YOD may miss out on the benefits of peer support, which could negatively impact their postdiagnostic experiences and social health.

A potential solution could be online peer support, such as support from social media, discussion forums, blogs, or video meetings, as it overcomes geographical barriers [25]. This benefit could make online peer support particularly useful for people who do not have access to local, age-appropriate peer support services or are unable to travel. Additionally, not everyone with YOD may feel ready to share their diagnoses and experiences with others. Online peer support allows people to engage in peer support from the comfort of their own homes, potentially lowering the barrier to join a peer support group. Finally, some people may experience challenges with speech due to their dementia symptoms. Online peer support can offer a variety of text-based (eg, social media and discussion forums) options, but it can also offer verbal (eg, video meetings) options for people who prefer those. Thus, online peer support could potentially make peer support accessible to a wide range of people and meet different needs and preferences.

Although previous studies showed how people with dementia use web-based platforms to connect with peers and exchange support, it remains unknown how users perceive this type of support, how such support impacts their daily lives, and what elements make online peer support meaningful [26-29]. Additionally, while previous research has been conducted into the experiences of facilitators of a variety of online peer support communities (eg, research by Coulson and Shaw [30] and Saha et al [31]), to our knowledge, this type of research has not been conducted in the field of dementia.

### Aims

Our study aims to develop (1) a best practice guidance on online peer support for people with YOD, so that people have access to evidence-based and tailored information about online peer support, and (2) best practice guidelines for facilitators of online peer support, so that they have access to tailored and evidence-based information that can be used to improve online peer support for people with YOD.

The study aims to answer the following research questions:

1. How do people with YOD use and experience online peer support?
2. What makes online peer support meaningful for people with YOD?
3. How can online peer support for people with YOD be improved?

## Methods

### Study Overview

**Figure 1:**
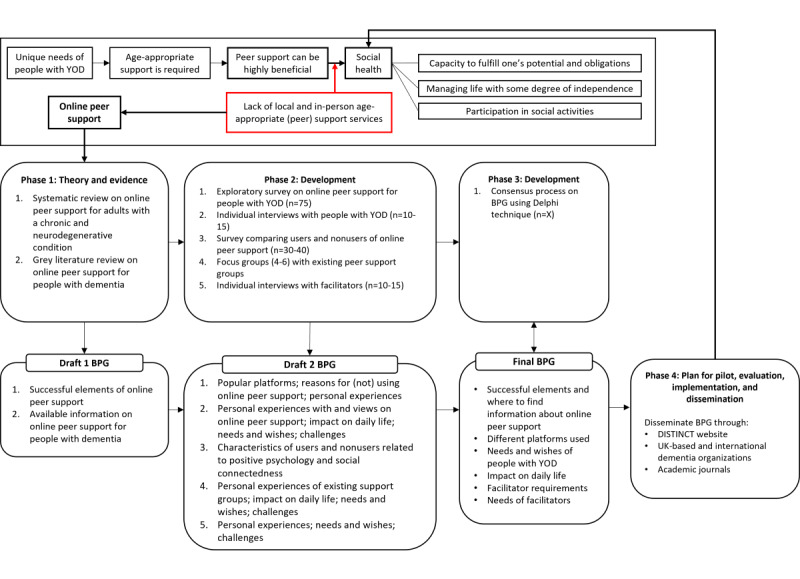
The development of best practice guidance on online peer support for people with YOD within the Medical Research Council framework. BPG: best practice guidance; DISTINCT: Dementia Intersectorial Strategy for Training and Innovation Network for Current Technology; YOD: Young Onset Dementia.

Our mixed methods study consists of 4 phases and follows the guidelines of the Medical Research Council on complex interventions [32]. The study focuses on the development stage of the Medical Research Council framework and develops a plan for the feasibility/piloting, evaluation, and implementation stages. Each phase consists of multiple substudies. Phases 1, 2, and 3 contribute to the development of the best practice guidance, including the guidelines for facilitators. Phase 4 consists of disseminating the best practice guidance and guidelines and developing a plan for a potential future pilot study, evaluation, and further implementation and dissemination. An overview of all 4 phases can be found in Figure 1.

### Ethics Approval

The study received ethical approval from the London Bromley Research Ethics Committee (reference number: 21/LO/0248).

### Informed Consent

All participants will provide informed consent before participating in any part of the study. Participants of a web-based survey will be asked to confirm that they have read and understand the study information and that they are happy to proceed before they can continue to the questions. Participants who fill in a paper copy or go through it verbally will be asked to do the same. The completion and submission of the survey will be taken as consent. For participants taking part in an individual interview or a focus group, consent will be taken remotely. This is due to the potentially wide range of geographic locations of participants and COVID-19 restrictions. There are 3 ways through which participants can provide consent, as follows: (1) signing a paper consent form and sending it back to the researcher, (2) signing a digital consent form and sending it back to the researcher, or (3) going through a verbal consent process with the researcher over a phone or video call. The members of the research team who are responsible for conducting the consent procedures have all undergone training on the Mental Capacity Act [33].

### Data Collection and Storage

The individual interviews and focus groups will be conducted remotely through Microsoft Teams, the group's usual meeting platform, or a phone call. The interviews and focus groups will be audio recorded with a University of Nottingham–approved recording device, and focus groups will be screen recorded via the videoconferencing platform. The recordings will be transcribed verbatim by a professional transcribing company that has an agreement with the University of Nottingham. Once the transcripts are completed, the recordings will be permanently deleted. The transcripts will leave out any information that could be used to identify a person. The recordings and transcripts will be stored on a password-secured, web-based storage space of the University of Nottingham.

### Patient and Public Involvement

During the design process of the study, people with YOD, carers, and health and social care professionals working with people with YOD were consulted. Senior members of the research team have extensive clinical experience in working with people with YOD and experience with patient and public involvement, cocreating research projects, and collaborating with people with dementia and their carers. Throughout the study, regular patient and public involvement consultations with people with YOD and health and social care professionals working with people with YOD will be conducted to discuss the progress of the study and study documents. All participants can receive the initial findings of the parts of the study in which they participated and provide further input on the analysis and findings before they get published.

### Phase 1: Theory and Evidence

The aim of this phase is to review the existing academic and grey literature on online peer support. The findings will set the foundation for the next phases and inform the first draft of the best practice guidance. This phase consists of the following two substudies: (1) a narrative synthesis systematic review on online peer support for adults with chronic neurodegenerative conditions and (2) a grey literature review on online peer support for people with dementia. Through the systematic literature review, successful elements of online peer support will be identified. Through Google searches and searches of websites of dementia organizations, the grey literature review will provide insights into the information that is available regarding online peer support for people living with dementia and how much of this information is tailored toward YOD.

### Phase 2: Development

#### Overview of Substudies

The aim of this phase is to identify the needs and wishes of people with YOD regarding online peer support and the kinds of information they need in the best practice guidance. This will be done by gathering experiences from people with YOD who use online peer support and those who do not. As such, this phase consists of the following five substudies: (1) an exploratory survey for people with YOD, (2) individual interviews with people with YOD, (3) a survey for comparing users and nonusers of online peer support, (4) focus groups with existing peer support groups for people with YOD, and (5) individual interviews with peer support facilitators. The findings of phase 2 will inform the second draft of the best practice guidance. An overview of each substudy in this phase is presented below.

#### Substudy 1

##### Exploratory Survey

A web-based survey will explore the different types of online peer support that people with YOD use, the benefits and challenges of different web-based platforms, and the positive and potential negative experiences and challenges that people may have undergone. Furthermore, the survey will explore why people do not engage in online peer support and identify potential barriers. This survey will be informed by the findings of phase 1 and set the foundation for the other substudies in this phase. The survey will be developed through Online Surveys (Jisc) [34] and will include multiple choice questions with the option to provide free-text responses. At the beginning of the survey, participants will answer questions on baseline characteristics (eg, age, gender, and time since diagnosis) and experiences with online peer support. At the end of the survey, participants will be asked if they would like to be involved in future parts of the study. Those who answer “yes” can provide their contact details. In this way, this substudy will be used as a pool for recruitment for the next two substudies (more details are provided in the *Substudy 2* and *Substudy 3* sections). Those who answer “no” can complete the survey anonymously.

##### Participants

People are eligible for the study if (1) they are living with a dementia diagnosis, (2) they received their diagnosis before the age of 65 years, and (3) they understand English. People do not have to be younger than 65 years at the time they take part in the survey. People living in a care facility will be excluded from the study because this population has daily contact with other people with dementia and is thus already involved in a form of in-person peer support, which can reduce the need and desire for remote, online peer support. Additionally, people living in a care facility are more likely to be in the more advanced stages of dementia, which is when the nature of symptoms can make it more difficult for people to use technology and engage in online peer support.

##### Recruitment

Participants will be recruited via convenience sampling through (1) National Health Service services, (2) dementia charities (eg, Dementia UK and Dementia Engagement and Empowerment Project [DEEP]), (3) dementia research networks (eg, Join Dementia Research and Rare Dementia Support), and (4) academic institutions (eg, the University of Nottingham). Furthermore, the survey will be advertised through social media and the professional network of the research team. The aim for the sample size is 75 participants, which is based on expertise within the research team.

##### Data Collection and Analysis

Participants can take part in the survey independently by following the link to the survey. Alternatively, they can receive a paper copy or go through the survey verbally with the researcher. This substudy will collect qualitative and quantitative data. The qualitative data will be analyzed by using thematic analysis, following the procedures outlined by Braun and Clarke [35], which consists of the following six phases: (1) familiarizing with the data; (2) coding the data; (3) developing initial themes; (4) developing and reviewing themes; (5) refining, defining, and naming the themes; and (6) writing up the findings. The analysis will be performed in NVivo (QSR International). The quantitative data will be analyzed in SPSS (IBM Corporation) by using descriptive statistics.

#### Substudy 2

##### Individual Interviews

The individual interviews with people with YOD will be used to build on the findings of substudy 1 and gather further insights into (1) reasons for engaging or not engaging in online peer support, (2) the impact of online peer support on daily life, (3) needs regarding online peer support, and (4) barriers to online peer support and how to overcome these barriers.

##### Participants and Recruitment

By using purposive sampling, a sample of the participants from substudy 1 who answered “yes” to the question about whether they would like to be involved in future parts of the study will be invited for an individual interview. The sample will be diverse in terms of baseline characteristics, the time since diagnosis, and experiences with online peer support.

##### Data Collection and Analysis

The individual interviews will be conducted in person or over a phone or video call, depending on the COVID-19 regulations, geographical locations of participants in relation to the researchers’ locations, and the participants’ preferences. The interviews will be audio recorded by using an external University of Nottingham–approved recording device and will be transcribed verbatim. The transcripts will be analyzed thematically via an inductive approach, following the procedures outlined by Braun and Clarke [35], and the analysis will be performed in NVivo.

#### Substudy 3

##### Survey Comparing Characteristics of Users and Nonusers

This survey compares users of online peer support with nonusers, aiming to identify characteristics of users and nonusers of online peer support. The aim is to further explore how online peer support impacts the daily lives of people with YOD and what it could provide to those who do not engage in (online) peer support. Participants will go through existing outcome measures related to social health and positive psychology, which will be informed by substudies 1 and 2. The survey will be developed through Online Surveys. Participants can go through the survey independently by following the link, receive a paper copy, or go through the survey verbally with the researcher.

##### Participants, Recruitment, Data Collection, and Analysis

Through purposive sampling, the pool of participants from study 1 will be used to select a sample from the participants who said “yes” to the opportunity to be involved in future parts of the project. The aim is to recruit 30 to 40 participants with an equal number of users and nonusers. To obtain 2 comparable groups, participants will be selected based on baseline characteristics. This substudy will collect quantitative data by using existing scales in the selected outcome measures.

#### Substudy 4

##### Focus Groups With Existing Peer Support Groups

This substudy consists of focus group interviews with existing peer support groups that have their meetings on the internet. During the COVID-19 pandemic, many support services for people with dementia have been disrupted and have had to move to the internet [36]. Videoconferencing platforms, such as Zoom (Zoom Video Communications Inc) and Microsoft Teams, have become more popular. The focus groups will be held on Microsoft Teams or the groups’ usual meeting platforms and will aim to provide insights into how people with YOD experience peer support through video meetings, how this experience impacts their daily life, and what the impact was of moving the meetings to the internet. This substudy will also explore the pros and cons of providing and receiving peer support through video meetings, the differences between in-person and online peer support, the potential challenges of online peer support, and how to overcome these challenges.

##### Participants

Participants will be subject to the same eligibility criteria as those in substudy 1, with the addition that they have to be part of an existing peer support group that meets on the internet or has experience with meeting on the internet. Groups do not have to be online-only groups; they are also eligible if they used to meet in person but moved their meetings to the internet during the COVID-19 pandemic.

##### Recruitment

Existing peer support groups will be recruited by using convenience and purposive sampling. With regard to convenience sampling, the study will be advertised through dementia charities (eg, Dementia UK and DEEP), dementia research networks (eg, Join Dementia Research and Rare Dementia Support), and academic institutions (eg, the University of Nottingham). Group facilitators and members can contact the research team if they are interested. With regard to purposeful sampling, the professional network of the research team will be consulted. The aim is to conduct 4 to 6 peer support groups, as data saturation tends to occur after 4 to 6 focus groups have been conducted [37]. The number of people in each focus group will depend on how many members of each peer support group want to take part.

##### Data Collection and Analysis

The focus groups will be screen and audio recorded by using the recording function of the videoconferencing platform and an external University of Nottingham–approved recording device, and the recordings will be transcribed verbatim. Additionally, the facilitator will take field notes. The transcripts will be analyzed thematically via an inductive approach, using the procedures outlined by Braun and Clarke [35], and the analysis will be performed in NVivo.

#### Substudy 5

##### Individual Interviews With Facilitators

The aim is to explore how facilitators of online peer support experience their role and what they believe is important. This will provide insights into (1) the role and tasks of a facilitator, (2) personal experiences of facilitating online peer support for people with YOD, and (3) challenges and how to overcome these challenges. The findings can contribute to a better understanding of how to facilitate online peer support for people with YOD.

##### Participants

People are eligible if they (1) are an online peer support facilitator, (2) are above the age of 18 years, and (3) speak and understand English. Someone will be considered a facilitator if they are responsible for setting up web-based meetings and facilitating such meetings or if they are facilitating or monitoring discussions on a text-based online peer support platform (eg, a Facebook group or a discussion forum).

##### Recruitment, Data Collection, and Analysis

With regard to the purposive sampling approach, peer support facilitators will be identified through substudies 1 and 4 and the professional network of the research team. With regard to snowball sampling, the researcher will ask participants if they know someone else who meets the eligibility criteria and would like to participate in the study. The aim is to conduct 10 to 15 interviews. This substudy will use the same data collection and analysis procedures as those in substudy 2.

### Phases 3 and 4: Development and Dissemination

#### Delphi Study

A draft of the best practice guidance and guidelines will be added to a web-based survey, which will consist of questions about content, format, readability, and dissemination. Those invited to take part in this study will be (1) everyone who took part in the study and said that they were interested in being involved in future parts of the study, (2) dementia charities (eg, Dementia UK and DEEP), and (3) professionals working with people with YOD. By using purposive sampling, participants from the previous substudies will be invited, and the survey will be shared through dementia charities, National Health Service services, and the professional network of the research team.

#### Consensus Meeting

A consensus meeting will be held to build on the findings of the Delphi study and gather input from study participants, supporters of people with YOD, and (health care) professionals working with people with YOD on the content and dissemination of the best practice guidance. A draft of the guidance will be shared with participants beforehand. Based on the outcomes of the meeting, the final guidance and guidelines will be developed.

The findings of the Delphi study and the consensus meeting will inform phase 4, during which a dissemination plan will be developed. The aim is to disseminate the best practice guidance and guidelines locally (in the United Kingdom) and internationally through dementia organizations and services, research networks, and academic institutions. Furthermore, a plan for a potential future pilot study that will test the best practice guidance and guidelines and for further implementation and dissemination will be developed.

## Results

Our study is funded by the European Union’s Horizon 2020 research and innovation program under the Marie Skłodowska-Curie Actions – Innovative Training Networks (H2020-MSCA-ITN-2018; grant agreement number: 813196). Phase 1 (theory and evidence) started in October 2019, and it is being updated throughout the study. In April 2021, the study received ethical approval from the London Bromley Research Ethics Committee (reference number: 21/LO/0248). Recruitment and data collection for phase 2 (development) started in May 2021. Data collection and analysis are expected to be completed by September 2022. Phase 3 (development) and phase 4 (dissemination) are expected to start in June 2022 and be completed by September 2022.

## Discussion

### Emerging Findings

Peer support can be a valuable source of support and make a positive impact on the postdiagnostic experiences of people with YOD [21,22]. This suggests that everyone living with YOD should have access to such support. Yet, research shows that many people with YOD experience difficulties with accessing age-appropriate (peer) support services [23,24]. Online peer support could be a solution. Although the nature of dementia symptoms can pose challenges to the use of technology, with the right guidance and support, many people can overcome such challenges and successfully use technology as a support tool in their daily lives. During the COVID-19 pandemic, many health and social care services have been disrupted and have moved to the internet, highlighting how important it is for people with dementia to be able to use technology [36]. However, for people with dementia to have better access to online peer support, the necessary guidance and support tools should be in place and be easy to access.

A best practice guidance on online peer support could raise awareness on the availability of this form of support, how to access it, and what it could provide to people. It could also help people to decide whether online peer support is something that could be helpful and, if so, which form of online peer support would be most suitable for them. Furthermore, best practice guidelines for facilitators will be developed to support them in improving online peer support and ensuring that such support meets the needs and wishes of people with YOD.

### Strengths and Limitations

The main strength of our study is that it addresses the inconsistent availability of health and social care services for people with YOD in the United Kingdom. Furthermore, the study includes people with YOD and health and social care professionals working with people with YOD throughout all phases. However, while online peer support overcomes geographical barriers and offers opportunities for international communication, the advertisement of and recruitment for the study will be conducted within the United Kingdom. Therefore, the findings will be specific to the UK context, and one should be cautious when generalizing the findings to other countries. Furthermore, some people may be unable to access online peer support for a variety of reasons, such as experiencing dementia symptoms that limit one’s ability to use technology or not having the (financial) resources for such support.

### Conclusions

People with YOD often experience different challenges compared to those of older adults with dementia and therefore need age-appropriate support. Peer support can contribute to a more positive postdiagnostic experience and every dimension of the social health framework. However, many people with YOD experience a lack of age-appropriate (peer) support services in their local area, indicating that online peer support could be a solution. Although research into online support for people with dementia is increasing, it remains unknown how users perceive this type of support, how such support impacts their daily lives, and what elements make it meaningful. Our study aims to explore how people with YOD use and experience online peer support and how online peer support can be improved. The findings will lead toward the development of a best practice guidance on online peer support that provides people with YOD with tailored and evidence-based information about online peer support. The guidance will also include guidelines for peer support facilitators who are aiming to improve existing online peer support opportunities and develop new online peer support opportunities.

## Abbreviations

DEEP: Dementia Engagement and Empowerment Project
YOD: young onset dementia

## Data Availability

Data sharing not applicable to this article as no data sets were generated or analyzed during the current study.
